# Beetroot Juice Supplementation as a Healthy Aging Strategy Through Improving Physical Performance and Cognitive Functions: A Systematic Review

**DOI:** 10.3390/nu17243954

**Published:** 2025-12-17

**Authors:** Anna Nowak, Angelika Szymańska, Magdalena Kwaśniewska, Ewa Kochan, Anna Lipert

**Affiliations:** 1 Department of Preventive Medicine, Medical University of Lodz, 92-213 Lodz, Poland; magdalena.kwasniewska@umed.lodz.pl; 2 Department of Pharmacological Biotechnology, Medical University of Lodz, 90-152 Lodz, Poland; angelika.gabrysiak@umed.lodz.pl (A.S.); ewa.kochan@umed.lodz.pl (E.K.)

**Keywords:** dietary nitrates, ergogenic nutrition, endurance capacity, aging physiology, cognitive outcomes

## Abstract

**Background:** Findings show that beetroot-derived nitrates can improve endurance, oxygen efficiency, muscular power, recovery and cardiovascular function, particularly in recreationally active or moderately trained individuals. However, results are mixed in elite athletes, likely due to their already optimized nitric oxide utilization. Cognitive function is a crucial aspect of athletic performance enabling athletes to adapt to dynamic environments and execute skills effectively, but evidence for cognitive benefits of nitrate-rich beetroot supplementation is limited and inconsistent. The combination of improved physical activity and cognitive functions contribute to overall healthy aging and extending life expectancy. This highlights the synergistic role of nutrition, exercise and mental agility in promoting long-term well-being. **Methods:** The literature review was conducted to summarize and systematize existing evidence on beetroot juice supplementation on physical performance and cognitive function in both, healthy adult population and athletes. **Results:** Overall, beetroot supplementation demonstrates strong potential as a natural ergogenic aid for enhancing physical performance, but current evidence on cognitive improvement remains inconclusive. **Conclusions:** Further research, particularly involving female or elite athletes, is needed to establish clear recommendations of beetroot juice supplementation as a supportive element of exercise capacity and cognitive abilities contributing to maintaining health and thus healthy aging.

## 1. Introduction

Nitrogen is a vital element for all living organisms. It’s main form, dinitrogen gas (N_2_), cannot be utilized by plants or animals. In order to be used by humans, it has to go through numerous processes in “the nitrogen cycle” [1,2]. The oral microbiome converts nitrates into nitrites. After ingestion, nitrate-rich saliva undergoes nonenzymatic metabolism in the stomach, producing nitric oxide (NO), which triggers various biological effects, e.g., neurotransmission, vasodilation or immunomodulation [3]. Nitrate (NO_3_^−^), along with nitrite (NO_2_^−^) were viewed as cancerogenic and adverse to the human diet [4,5]. Both NO_3_^−^ and NO_2_^−^ have been one of the ingredients added during the process of curing certain meat products to act as a preservative against microorganisms responsible for food poisoning [6]. With today’s knowledge we can say that nitrates have a wide range of positive effects on the human body, such as: lowering blood pressure, increasing flow-mediated dilation, reducing platelet–monocyte aggregates and improvement in vascular function [7].

Nitrate, nitrite and N-nitroso-compounds can be synthesized endogenously. In healthy individuals, approximately 1 mmol (62 mg) of nitrate is produced per day. This endogenous production becomes particularly significant when dietary nitrate intake is low or during gastrointestinal infections that affect the stomach pH [8]. Following a comprehensive re-evaluation of their safety, the European Food Safety Authority (EFSA) has concluded that the established safe levels for nitrites and nitrates intentionally added to meat and other food products provide adequate protection for consumers. Nitrite and nitrate salts (such as sodium and potassium salts; labelled on food products as: nitrites E 249–250 and nitrates E 251–252) are approved food additives in the EU. They are used for various reasons, for example to protect products such as: meat, fish, and cheese, from microbial growth or to preserve color and flavor. The current acceptable daily intake (ADI) is 3.7 mg/kg body mass/day for nitrates and 0.07 mg/kg body mass/day for nitrites [9,10]. For humans, the lethal oral dose for nitrate can range from 67 to 833 mg/kg body mass. When it comes to nitrite the lethal dose, it ranges from 33 up to 250 mg/kg body mass [8].

**Figure 1:**
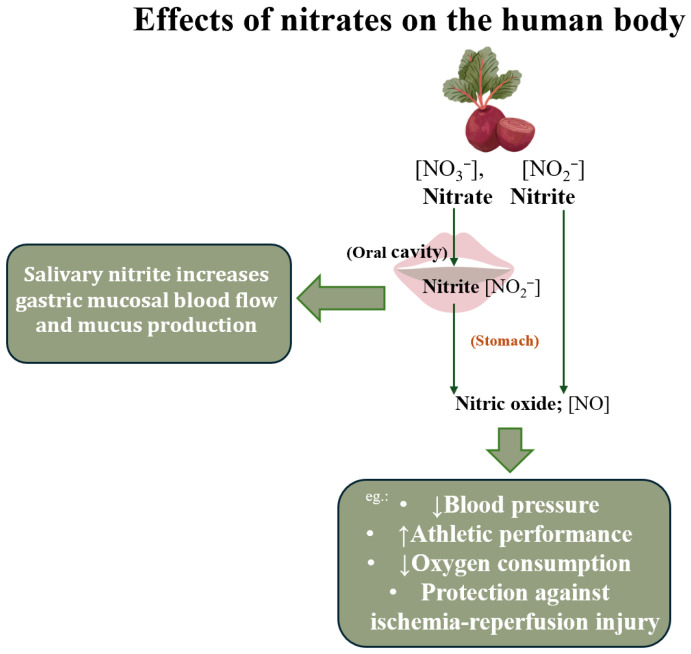
The effects of nitrates on the human body.

Vegetables are the primary source of nitrate, contributing to about 80–85% of the daily nitrate intake and up to 43% of nitrite intake [6,11]. Nitrates can be found in a variety of vegetables and their nitrate concentration can differ due to factors such as: weather conditions, soil quality and its pH or the plant species [12]. Greatestnitrate levels (>2500 mg/kg), according to their nitrate content, are vegetables and leafy greens like spinach, lettuce and beetroot, which is both a root and a leaf crop. Beetroot is grown for its edible storage roots and leaves (mainly young) [13]. Due to its great nitrates levels, it has gained popularity among athletes and is the most commonly used form of supplementing dietary nitrate. It can enhance exercise performance through multiple pathways, including vasodilation, improving blood circulation, improving muscle and cerebral blood flow, lowering oxygen demand in skeletal muscles or decreasing the buildup of anaerobic respiration byproducts which helps to not only boost power output and muscle force but also delay fatigue (Figure 1) [7].

The concentrations of nitrate (NO_3_^−^) and nitrite (NO_2_^−^) in beetroot supplements can vary widely due to differences in beetroot sourcing, processing methods, storage conditions and addition of other compounds. Since these compounds are sensitive to factors like heat, light, and time, their levels can degrade or fluctuate during production and shelf life. Unfortunately, not all manufacturers standardize their products for nitrate or nitrite content, leading to significant disparities between brands or even batches of the same product. As a result, the actual nitrate and nitrite content in beetroot supplements may differ from what is expected or labelled [14,15].

Studies have shown that dose-dependent increase of plasma nitrate and nitrite and a reduction in the oxygen cost of moderate-intensity cycling, were noted at the same supplemented dose being ~16.8 mmol. However, lower doses of nitrate supplements were the ones to improve the time-to-task failure during severe-intensity exercise [16]. Additionally, individuals with very great levels of aerobic conditioning, particularly elite endurance athletes (with a VO_2_max above 65 mL·kg^−1^·min^−1^), generally do not experience significant performance gains from nitrate supplementation [17,18]. Nowadays, the protocols of nitrate supplementation in sports, recommend the dose of ~6-6 mmol (350–500 mg), taken approximately around 2–3 h before planned exercise [19].

Maintaining a nutritious diet and achieving optimal nutritional balance are key strategies for supporting brain function and cognitive well-being [19]. Cognitive abilities refer to several interconnected mental operations—such as our capacity to retain and recall information (memory), the ability to concentrate and filter distractions (attention), greater level planning and self-regulation (executive functions), problem-solving, and the speed of mental processing [20]. Research suggests, that NO_3_^−^ supplementation may positively impact these abilities. Benefits were seen in a few age-groups, including healthy young individuals, both at rest and during exercise, particularly after 5–7 days of supplementation. During intense exercise—when brain oxygen drops—cognition may suffer, and NO_3_^−^’s benefits appear limited. Yet, higher doses (~13 mmol) over 7 days improved decision-making during prolonged, high-intensity intermittent exercise [21].

Constantly growing evidence shows that supplements containing natural extracts, pure extracts, or juices from plants can have a positive effect and stimulate cognitive performance in humans. Peth-Nui et al. 2012, demonstrated that the use of *Bacopa monnieri* improves concentration and memory processing, as well as contributing to increased working memory, and even that the use of this plant extract may be useful in the treatment of attention deficit disorder [22]. Similar observations were related to the extracts derived from the *Panax ginseng* plant [23]. In addition, it was proved that ginseng’s supplementation had a beneficial effect on cognitive impairment caused by Alzheimer’s disease [23]. Other adaptogenic plant, *Rhodiola rosea* L., significantly improved reaction and choice times in healthy men in the study by Stojcheva et al. 2022 [24]. What is more, taking Ashwagandha extract resulted in improved memory and concentration, which classifies it as an effective adaptogen, improving cognitive abilities, as shown in a study by Gopukumar et al. 2021 [25]. In a similar manner, beetroot juice also deserves recognition for its cognitive benefits. Heiland E. et al. 2024, demonstrated that supplementing beetroot juice resulted in improved cognitive parameters, as study participants achieved significantly better results in serial subtraction task than those receiving a placebo [26]. Gilchrist et al. 2014, observed that two weeks of beetroot juice supplementation resulted in significant improvements in simple reaction time in individuals with type 2 diabetes [27]. In a study by Vaccaro et al. 2024, ingesting a beetroot-based supplement improved cognitive function, particularly memory capacity and frontal nerve function [28].

**Figure 2:**
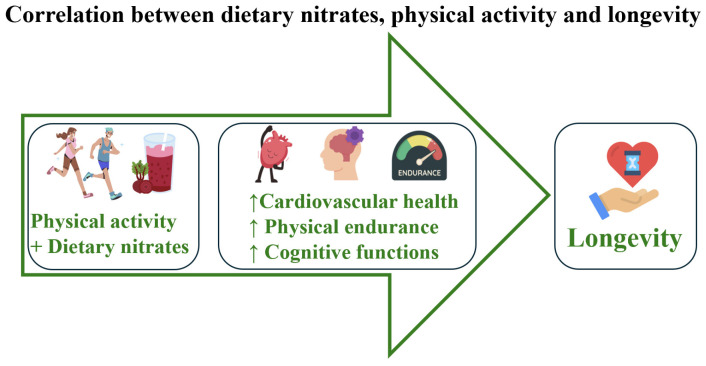
The correlation between dietary nitrates, physical activity and longevity.

An increasing number of studies shows that both physical performance and endurance are strongly associated with cognitive functions. A recent umbrella review and meta-meta-analysis of 133 included studies found that physical exercise significantly improves global and executive functions. Importantly, the study highlighted that light- to moderate-intensity activities and physically interactive games (exergames) may be especially effective in enhancing mental performance [29]. Shorter reaction times and executive functions can translate into better athletic performance. Such observations were made by Trecroci et al. 2021, who demonstrated that young female volleyball players whose basic cognitive functions were better developed demonstrated better physical fitness in a given discipline than those whose cognitive functions were at a lower level [30]. Dietary nitrate supplementation, when combined with regular physical activity, can further enhance exercise efficiency by improving muscle oxygenation and endurance. Together, these effects create a synergistic benefit, promoting both cardiovascular health and extended lifespan (Figure 2). In the context of healthy aging, evaluating physical activity and cognitive function in young to middle-aged adults is crucial, as this stage represents the peak of physiological capacity and cognitive performance. Maintaining regular physical activity during adulthood supports cardiovascular, metabolic, and musculoskeletal health—fundamental components in the prevention of chronic disease and lowering mortality risk. Furthermore, improving cognitive functions reduces the risk of neurodegenerative disorders later in life [31].

This literature review was conducted to summarize and systematize existing evidence on beetroot juice supplementation on both physical performance and cognitive functions in healthy adult population as well as athletes. By identifying the significant variations in dosing strategies, nitrate concentrations, supplementation durations, and practical applications used across studies, we highlight how these methodological differences influence the outcomes relevant to functional capacity in older adults. This review points out the lack of a standardized beetroot juice supplementation protocol and the integration of these two areas of research. This issue currently limits the development of a clear, evidence-based guidelines for its use as a healthy aging strategy and emphasizes the need for consensus in future research. We believe that properly used supplementation with beetroot juice at a younger age, which supports exercise capacity and cognitive abilities, can contribute to maintaining health and thus healthy aging. Therefore, this paper aims to bridge this gap by examining the dual role of beetroot juice in supporting both cognitive functions and physical performance and exploring its use as a strategy for healthy aging.

## 2. Materials and Methods

### 2.1. Search Strategy

This literature review was conducted to assess the effects of beetroot juice and nitrates supplementation on cognitive function and physical performance in humans. The inclusion criteria for the review contained (a) studies conducted on humans, (b) studies conducted using beetroot and/or plants containing nitrates, (c) studies assessing cognitive functions and/or physical activity.

A literature review covering the years 2020–2025 was conducted to assess the impact of nitrate-rich beetroot supplementation on physical performance and cognitive function in healthy individuals, including both the general population and athletes aged 18–59. The search strategy was based on the use of combinations demonstrated in Table 1. The analysis included both experimental studies and review articles, allowing for the collection and critical analysis of available research findings. The aim of this study was not only to summarize existing reports and to systematize them in a way that enabled a synthetic presentation of the most current knowledge on the potential ergogenic and neurocognitive effects of beetroot supplementation in the study group, but also to indicate insufficient data for future research.

### 2.2. Exclusion Criteria

The first exclusion phase gathered publications on humans suffering from any chronic diseases and symptoms such as hypertension, respiratory infections, diabetes, menopause etc. Other excluded papers involved addiction and various substances use such as alcohol, drugs, smoking and others. Second exclusion phase focused on publications duplicated in both databases and, therefore, were rejected.

### 2.3. Search Results

**Figure 3:**
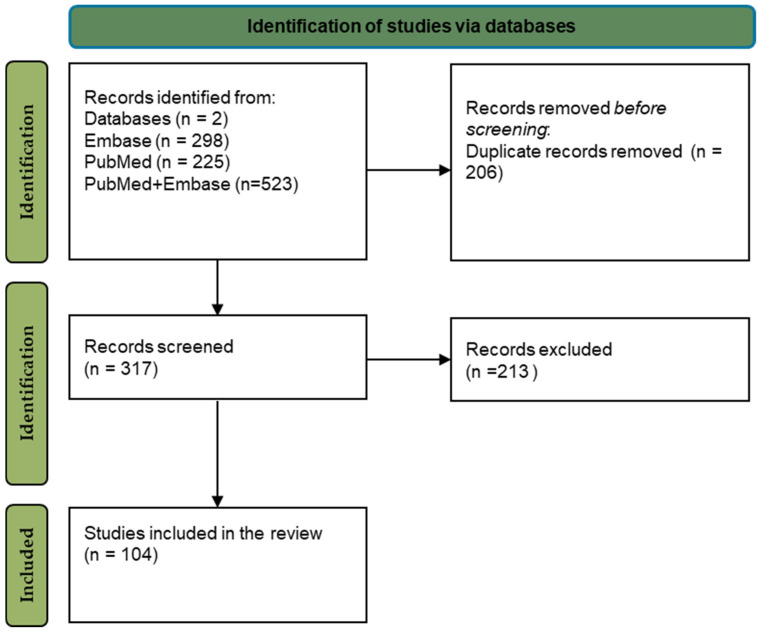
PRISMA flow diagram of the study selection process.

This review followed the guidelines of the Preferred Reporting Items for Systematic Reviews and Meta-analyses (PRISMA). Figure 3 illustrates a PRISMA flow diagram of the study selection process for all articles. The PRISMA checklist is listed as Table S1.

All search strategies of the two databases yielded a total of 523 articles. Based on the full-text analysis, 213 records were excluded. There were 206 articles repeated in Embase and PubMed. Search terms in the electronic database Embase yielded a total of 298 matching results. A search of the electronic database PubMed yielded a total of 225 records. After subsequent comparison with the Embase database, as many as 206 results that also appeared in this database were excluded. Some articles were excluded after the screening phase because the presentation of the data did not match the inclusion criteria. In total 104 studies were selected in the present review.

## 3. Results

This review analyzed data on the effects of nitrates from various forms of beetroot (juice, extracts in capsules, etc.) on physical activity and cognitive function. Table 2, Table 3 and Table 4 summarize the results based on experimental articles. Table 5 includes information from review articles and meta-analyses.

### Analysis of Nitrates on Physical Activity and Cognitive Functions (Original Articles)

Studies shown in Table 2 and Table 3 reported ergogenic effects of dietary nitrate (primarily sourced from beetroot) on exercise performance and cognitive functions. Endurance outcomes have improved in several studies—for example, Huang et al. observed longer time-to-exhaustion in cyclists [32], and Tirkey et al. found ~4–8% faster 10 km run times after supplementation [33]. Improvements in power and muscle strength were also noted, e.g., Rodríguez-Fernández et al. reported increased peak and mean power output during resistance exercise [34], while Jurado-Castro et al. showed greater: jump height, lifting velocity, and repetitions to failure after beetroot juice supplementation [35]. A few studies indicated enhanced recovery manifested by quicker recovery of the muscle function with less perceived soreness [36] as well as faster post-exercise cardiovascular recovery showed by Benjamim et al. [37]. In contrast, other investigations found no significant changes after nitrate supplementation (Table 4). For instance, in a study by Tan et al. no improvements in sprint, strength, or aerobic measures among female team-sport athletes were spotted. Similar observations had Burke et al.—this study reported no effect on endurance performance or exercise efficiency in elite race walkers [38]. López-Samanes et al. and Trexler et al. observed no gains in power, speed, or overall exercise performance with nitrate supplementation [39,40]. Unfortunately, only one trial showed positive visible effect of supplementing nitrates on cognitive functions. Improved cognitive performance on a Stroop-test after a moderate (400 mg of NO_3_^−^) nitrate dose was noted by Miraftabi et al. [41].

The analyzed studies provide overall support for the beneficial effects of beetroot supplementation on physical activity. However, inconsistencies might be due to differences in, for example: test sensitivity, participants’ fitness levels or sample size.

Several trials reported improved endurance and oxygen efficiency. Beetroot supplementation has shown consistent effects in improving cardiovascular health and reducing exercise-related stress. Numerous studies reported reductions in heart rate and perceived exertion by lowering heart rate and exertion during work intervals in women or decreasing ratings of perceived exertion and muscle soreness after functional tests at altitude.

Beetroot-derived nitrates also appear to improve aerobic capacity and endurance in a variety of training situations. A growing body of literature demonstrates improved VO_2_max and ventilatory efficiency in women [42] and achieving similar gains in VO_2_max and rowing performance [43]. Certain studies have observed enhanced 10-km running time trial performance in both men and women, suggesting benefits across endurance modalities [33]. However, acute beetroot juice ingestion did not improve sprinting, strength, or aerobic performance in female team-sports [44], nor did it enhance tennis-specific performance in elite players [45]. Similarly, studies in trained and endurance athletes [46,47] reported no improvements, despite elevated plasma nitrate/nitrite concentrations. These mixed results may be explained by the fact that elite athletes are already very efficient at using nitric oxide, leaving little room for further improvement. As highlighted by one of the analyzed systematic reviews, the benefits of beetroot supplementation seem to be greater in individuals who are less trained [48].

Several studies highlight beetroot’s potential in resistance exercise. Parameters such as increased repetitions to failure, power, and velocity, as well as improved resistance outcomes have been reported [35,49]. Higher doses of beetroot supplementation led to greater improvements in muscle torque development, while supplementation also increased resistance to fatigue during repeated knee extensions. Together, these results indicate that beetroot supplementation can enhance both the efficiency of muscle contractions and overall endurance during resistance exercise [50,51].

When considering studies used in this systematic review, most used nitrate doses between 6 mmol and 13 mmol, usually delivered in a beetroot juice or a concentrate form, consumed 2–3 h before exercise. Some of the trials tested lower doses (~4–6 mmol) and still observed some improvements in heart rate, oxygen cost, or muscular endurance [52]. Worth mentioning is the fact that higher doses (>15 mmol) did not provide clear additional benefits [50].

**Table 2:** Summary of the original studies examining the impact of dietary nitrates from beetroot on exercise performance—acute dosing.

	Positive Effects of Supplementation (Acute/Short-Term)					
	Study	Sample Size	Dose	Form	Duration	Results
1	Ahmadpour A. et al. [53]	10 men	PLA (<0.5 mmol NO_3_^−^) or BRJ 220 mL (~8.9 mmol NO_3_^−^) consumed 2.5 h before functional tests at 2800 m altitude.	Juice	Acute	Beetroot juice (BRJ) improved isometric muscle endurance (wall-sit), anaerobic capacity (90 s box jump, BJ90), agility (Hex Jump), and reduced time to change directions. BRJ also lowered ratings of perceived exertion (RPE) during slalom (SL)runs and muscle soreness (MS) at 12, 24, and 48 h post-exercise.
2	Benjamim C.J.R. et al. [37]	16 men	Beetroot extract (600 mg capsule) vs. placebo, taken 120 min before exercise. The participants ingested the opposite intervention (placebo or beetroot extract) on the third and final day to guarantee the study’scross-over.	Capsules	Acute (3 days; crossover).	Beetroot extract accelerated recovery—systolic blood pressure (SBP) and diastolic blood pressure (DBP) returned to baseline faster, heart rate (HR) recovered more quickly (elevated only 0–5 min vs. 10 min in placebo), and heart rate variability (HRV; HF index) recovered earlier.
3	Black M.I. et al. [54]	11 individuals (10 men; 1 woman)	7-day low NO_3_^−^ diet, 3-day high NO_3_^−^ diet, compared with a standard (control) NO_3_^−^ diet;	Nitrates from food	Short-term dietary interventions: 7 days low NO_3_^−^, 3 days high NO_3_^−^, with controlled washout periods.	Low NO_3_^−^—reduced saliva [NO_3_^−^] and [NO_2_^−^] and plasma [NO_3_^−^]. High NO_3_^−^ intake: improved sprint cycling peak power output (+4%) and mean power output (+3%), increased saliva and plasma [NO_3_^−^]/[NO_2_^−^] more when preceded by a low NO_3_^−^ diet; BP was reduced following high NO_3_^−^ intake when preceded by a standard diet;
4	Bloomer R.J. et al. [55]	10 men and 10 women	RRB1: Resync Recovery Blend, 1 serving (~7.5 g; ~4.2 g nitric oxide blend), single acute ingestion mixed with 12 fl oz water; RRB2: Resync Recovery Blend, 2 servings (~15 g; ~8.4 g nitric oxide blend), single acute ingestion mixed with 12 fl oz water; RCB1: Resync Collagen Blend, 1 serving (~21 g; ~2 g proprietary blend), single acute ingestion mixed with 12 fl oz water; PLA: Placebo, 7.5 g nitrate- and polyphenol-free powder mixed with 12 fl oz water.	Drink/juice	Acute	Resync supplements raise plasma NO_x_, especially the Recovery Blend in a dose-dependent way, without affecting heart rate or blood pressure.
5	Cocksedge S.P. et al. [56]	10 men	Nitrate-rich beetroot juice concentrate (210 mL containing ~18.6 mmol NO_3_^−^); 2.5 h before exercise on each testing. each trial was conducted on nine occasions over a 4–7 week timeframe, with beetroot (BR) or placebo (PL) consumed 2.5 h prior to each exercise test under normoxia, hypoxia, or hyperoxia.	Juice	Acute	Acute BRJ (~18.6 mmol NO_3_^−^) increased plasma [NO_2_^−^] and quadriceps oxygenation during moderate-intensity cycling. During severe-intensity cycling, BR enhanced exercise tolerance in hypoxia but not in normoxia or hyperoxia, with greatest benefits observed in individuals experiencing higher skeletal muscle deoxygenation.
6	De Souza D.B. et al. [57]	20 men	Beetroot juice (BJ; 500 mL, 16 mmol NO_3_^−^); 60 min before exercise; six exercise or PLA (açaí-flavored maltodextrin, equalized the caloric content of the BJ + 20 mL of beetroot to give flavor of the PLA)	Juice	Acute	Acute BJ supplementation significantly decreased 4-km running time in both concurrent training sessions (CT1: aerobic + resistance; CT2: resistance + aerobic) compared to placebo and control. Plasma nitric oxide concentration increased after BJ, while placebo showed no significant change.
7	Dumar A.M. et al. [58]	10 men	Single dose of 70 mL concentrated beetroot juice (~400 mg NO_3_^−^), consumed 2 h prior to exercise. Participants consumed the full dose within 5 min; PLA (blackcurrant juice)	Juice	Acute	Supramaximal exercise performance (Wingate tests, 3 × 15 s) was lower in the morning with PL (AM-PL) compared to afternoon (PM) and morning BRJ (AM-BRJ). Mean power, anaerobic capacity, and total work were all significantly higher in AM-BRJ vs. AM-PL and similar to PM. Heart rate was lower in AM-BRJ than AM-PL. RPE—RPE was significantly lower during WAnT1 (Wingate anaerobic test) than for WAnT2 (*p* = 0.002) and WAnT3 (*p* < 0.001). Acute BRJ ingestion mitigates the reduction in AM supramaximal exercise performance, resulting in performance comparable to PM levels, with lower HR but unchanged RPE.
8	Esen O. et al. [59]	12 men	NO_3_^−^-rich beetroot juice (NIT, 140 mL, ~12.8 mmol NO_3_^−^) or NO_3_^−^-depleted placebo (PLA, 140 mL, ~0.04 mmol NO_3_^−^), consumed 3 h before the Yo–Yo IR1 test.	Juice	Acute	Acute high-dose NO_3_^−^ supplementation with beetroot juice significantly enhanced intermittent running performance in the Yo–Yo IR1 test.
9	Forbes S. et al. [60]	14 women (including 9 using hormonal contraceptives; HC)	Nitrate-rich beetroot juice (140 mL containing ~13 mmol NO_3_^−^; 2,5 h before exercise or PLA (NO_3_^−^-free blackcurrant juice)	Juice	Acute	BRJ did not reduce VO_2_ or heart rate at 50% and 70% VO_2_max in the majority of participants, though 3 participants were responders showing ≥3% VO_2_ reduction. Rate of perceived exertion (RPE) was significantly reduced with BRJ compared to placebo, especially at higher intensity (70% VO_2_max)
10	Garnacho-Castaño M.V. et al. [61]	10 men	BRJ—140 mL (~12.8 mmol, ~808 mg NO_3_^−^), consumed 3 h before the 2000-m rowing ergometer test; or PLA (made by dissolving 2 g of powdered SUPER BEETROOT (~0.01 mmol, 0.620 mg of NO_3_^−)^ in 1 L of water))	Juice	Acute	Possibly improved 2000-m time trial performance (mean difference 4 s) Increased relative and absolute VO_2_ max (mean difference 2.10 mL·kg^−1^·min^−1^ and 0.16 L·min^−1^).
11	Garnacho-Castaño M.V. et al. [43]	11 men	Beetroot juice (BJ, 140 mL, ~12.8 mmol NO_3_^−^/808 mg) administered 3 h before exercise; or PLA (prepared by dissolving 2 g of powdered BJ (~ 0.01 mmol, 0.620 mg of NO_3_^−^)	Juice	Acute	Plasma NOx levels after BJ: reduced pulmonary oxygen uptake (VO_2_) during rest and full back squat exercise. BJ: plasma myoglobin levels increased;
12	Garnacho-Castaño M.V. et al. [62]	12 men	Beetroot juice (140 mL; ~12.8 mmol NO_3_^−^ (~808 mg)); 3 h before exercise (of each test) or PLA (prepared by dissolving 2 g of powdered BJ (~ 0.01 mmol, 0.620 mg of NO_3_^−^)	Juice	Acute	Acute BJ (~12.8 mmol NO_3_^−^) enhanced CrossFit WOD (short and high intensity daily sessions) performance when rest periods were included between exercises, but also increased muscle fatigue, cortisol, and arterial desaturation.
13	Hemmatinafar M. et al. [63]	12 women	Beetroot juice (BRJ) vs. placebo (PLA), 50 mL per serving, 8 servings over 2 days (total 400 mL), ingested at 2, 6, 10, 14, 26, 30, 34, and 38 h post-exercise.	Juice	Acute (2-days)	BRJ improves static muscular endurance performance (in female volleyball players) 48 h after exercise-induced muscle damage, which is also associated with reducing the perception of muscle soreness and tissue edema.
14	Jiaqi Z. et al. [52]	13 women	BJ 2.5 h before exercise; single dose 70 mL (~6.45 mmol NO_3_^−^) or double dose (2 × 70 mL; ~12.9 mmol NO_3_^−^). or PLA (BJ with extracted nitrates)	Juice	Acute	Decreased mean heart rate (HR) and ratings of perceived exertion (RPE) during work intervals, recovery periods, and across the overall protocol in women; no additional benefits were observed with the higher 12.9 mmol dose.
15	Jurado-Castro J.M. et al. [64]	11 men	70 mL beetroot juice (BJ; 400 mg NO_3_^−^, 6.4 mmol/L or NO_3_^−^-depleted placebo, consumed 120 min before resistance training sessions.	Juice	Acute	BJ enhanced performance in resistance training tests, improving the number of repetitions completed. Max HR higher in BJ compared to placebo. Root Mean Square of the Successive Differences (RMSSD) decreased during exercise with BJ (*p* = 0.023; ES = 0.999), indicating greater parasympathetic withdrawal during activity. RMSSD-Slope (internal load indicator) improved with BJ (BJ: 3 ± 3 vs. placebo: 0.5 ± 0.7; *p* = 0.025; ES = 1.104), suggesting lower internal load despite better performance. Acute BJ supplementation 120 min before resistance training reduces internal load during exercise (improved RMSSD-Slope) while enhancing muscular endurance performance.
16	Jurado-Castro J.M. et al. [35]	14 women (2 on contraceptives)	70 mL NO_3_^−^-rich beetroot juice (BRJ; 400 mg nitrate) or NO_3_^−^-depleted placebo, consumed 2 h before exercise (during each visit—3 visits)	Juice	Acute	Greater maximum height in countermovement jump (CMJ) after BRJ compared with PLA (+6%) Increased mean velocity, peak velocity mean power and peak power; Higher repetitions to failure (RTF) in back squat, leg press, and leg extension exercises; significant main effects for time, supplement, and supplement × time interaction in all exercises.
17	Macuh M. et al. [65]	15 men	70 mL concentrated beetroot juice (~400 mg nitrate)) or nitrate-depleted placebo (~0 mg nitrate), consumed 2 h before exercise	Juice	Acute	Running performance (Cooper test) improved significantly with nitrate supplementation when baseline dietary nitrate intake was <300 mg/day (+0.145 km on average).
18	Miraftabi H. et al. [41]	8 men	four experimental trials: BJ-400, BJ-800, PL, CON; 2.5 h before the tests, each participant ingested either one bottle of 60 mL BJ + one bottle of 60 mL of PL or two bottles of BJ (120 mL) (=400 mg NO_3_^−^ per bottle) or depleted dried powder NO_3_^−^ for PL (1 g of dried powder of BJ dissolved in 1 L of water + lemon juice for taste)	Juice	Acute	Moderate to large effect sizes on anaerobic performance and a large effect size on aerobic performance, BJ-400 improved cognitive function (word–color and total scores).
19	Neteca J. et al. [42]	18 women	BJG- 50 mL of nitrate-rich beetroot juice concentrate (~6.2 mmol of nitrates (NO_3_^−^) consumed once before second exercise test; or PLA (nitrate-free beverage).	Juice	Acute	VO_2_ max increased by 4.82%, ventilation efficiency (VE/VO_2_ and VE/VCO_2_) improved; Heart rate decreased, indicating enhanced cardiovascular and respiratory function and reduced fatigue during prolonged exercise.
20	Ranchal-Sanchez A. et al. [66]	12 men	Beetroot juice (70 mL containing ~400 mg NO_3_^−^ per serving); 120 min before exercise, single acute dose, across three visits; or PLA (blackcurrant juice with depleted nitrates)	Juice	Acute	Acute BJ (~400 mg NO_3_^−^) enhanced muscular endurance in back squat and overall session repetitions.
21	Rodríguez-Fernández A. et al. [34]	18 men	Beetroot juice (BJ), 140 mL total (2 × 70 mL concentrated shots; ~800 mg NO_3_^−^otal); ingested 2.5 h prior to testing or PLA (2 × 70 mL providing <0.1 mmol NO_3_^−^)	Juice	Acute	Mean power (MP): BJ increased MP during both concentric (CON) and eccentric (ECC) contractions across moment inertias of 0.025, 0.050, 0.075, and 0.100 kg/m^2^. Peak power (PP): BJ increased peak power during both CON and ECC contractions across all moment inertias; Total power output increased at all moment inertias with BJ; improvements were similar for CON and ECC contractions (15–25% improvement). Interpretation: Acute BJ ingestion enhanced skeletal muscle contractile function and power output during half-squat exercise at multiple inertial loads.
22	Rowland S.N. et al. [67]	12 men	Beetroot juice 2 × 70 mL (~13 mmol NO_3_^−^) with breakfast 2.5 h before exercise at morning (08:00), afternoon (12:00), or evening (15:00). Six experimental conditions, PL and BR in the morning (started at 08:00; PL-MORN and BR-MORN), afternoon (started at 12:00; PL-AFT, BR-AFT) and evening (started at 15:00; PL-EVE and BR-EVE).	Juice	Acute	Central systolic BP was reduced 2.5 h after BR ingestion at all timepoints. BR increased salivary and plasma [NO_3_^−^] and [NO_2_^−^] similarly across morning, afternoon, and evening.
23	Serra-Payá N. et al. [68]	11 men	140 mL Beet-It-Pro Elite Shot (~808 mg NO_3_^−^ (~12.8 mmol)), 3 h prior to testing or PLA (2 g of powdered BJ (~0.01 mmol, 0.620 mg of NO_3_^−^, dissolved in 1 L of water + lemon juice for flavor)).	Juice	Acute	Acute BJ intake improves FS (full squad) resistance exercise performance, ventilatory efficiency (VE/VCO_2_ slope), and PetCO_2_ when rest is provided between exercises, likely due to enhanced NO_3_^−^ → NO_2_^−^ → NO conversion and pulmonary vasodilation. The effect is not observed under severe anaerobic conditions without rest.
24	Tan R. et al. [69]	14 men	2 × 70 mL doses per day of concentrated NO_3_^−^-rich beetroot juice (BR: ~5.9 mmol of NO_3_^−^ per 70 mL). Experimental days (day 1 and 4 of each supplementation period): 2 × 70 mL of allocated beverage 2.5 h before exercise. Days 2 and 3 of each supplementation period: 1 × 70 mL beverage twice a day; or PLA (nitrate-depleted BR)	Juice	2 × 4 days	Acute BRJ supplementation increased upper-body muscular endurance (bench press RTF) and raised plasma [NO_3_^−^].
25	Tatlici A. et al. [70]	8 men	Second visit of the trial: 2 × 70 mL beetroot juice; Third visit of the trial: 2 × 70 mL beetroot juice, 150 min prior to testing or PLA (140 mL of cherry + lemon juice).	Juice	Acute	Balance performance after fatigue-inducing full-squat exercise (FSE) was significantly better in the BRJ group compared to placebo. At rest, some balance parameters were also improved in the BRJ group. Static Medial-Lateral Stability Index (MLSI) improved significantly with BRJ. Dynamic OSI (overall stability index) and APSI (anterior/posterior stability index) were improved with BRJ. Post-fatigue, BRJ supplementation improved Static OSI (ES = 1.3), APSI (ES = 1.2), Dynamic OSI (ES = 1.1), APSI (ES = 1.0), and MLSI (ES = 1.0) compared to placebo. Acute BRJ ingestion improves balance performance both at rest and following fatigue in trained wrestlers
26	Tatlici A. et al. [71]	8 men	Beetroot juice (BJ), 2 × 70 mL shots (~140 mL), 150 min before exercise or PLA (140 mL of cherry + lemon juice).	Juice	Acute	Acute BJ supplementation significantly increased upper-body (shoulder) strength and average lower- and upper-body strength
27	Thurston T.S. et al. [72]	11 men	Single daily dose for 2 days prior to experimental trial, plus a double dose 2 h before exercise (Nitrate-rich beetroot concentrate; 70 mL; 4.1 mmol NO_3_^−^) or PLA (nitrate-stripped; 0.03 mmol of NO_3_^−^).	Juice	Short term (3-day supplementation period; single doses for 2 days and double dose 2 h prior to exercise.)	Acute nitrate-rich beetroot concentrate supplementation elevates plasma nitrate and nitrite, lowers mean arterial pressure (MAP) and leg blood flow during cycling.
28	Volino-Souza M. et al. [73]	9 women and 4 men	Beetroot juice (BJ), 140 mL containing ~8.12 ± 3.61 mmol NO_3_^−^; consumed 150 min before exercise or PLA (depleted nitrate beetroot juice; ~0.08 ± 0.76 mmol of nitrate).	Juice	Acute	A single acute dose of BJ (~8.1 mmol NO_3_^−^) enhanced muscle reoxygenation during recovery
29	Wei C. et al. [50]	8 men and 3 women	2 bottles of NO_3_^−^ depleted BR (placebo, PL) (~0.08 mmol NO_3_^−^, 2 × 70 mL); 1 bottle of NO_3_^−^ rich BR (~6.4 mmol NO_3_^−^, 1 × 70 mL); 2 bottles of NO_3_^−^− rich BR (~12.8 mmol NO_3_^−^, 2 × 70 mL); 3 bottles of NO_3_^−^− rich BR (~19.2 mmol NO_3_^−^, 3 × 70 mL); 1.3 g KNO3 (~12.8 mmol NO_3_^−^ mixed with 300 mL deionized water on separate laboratory visits.	Juice	Five visits over a period of 17–35 days	Muscle contractile function showed dose-dependent effects: mean peak torque and torque impulse were significantly enhanced during the first 10 muscle contractions following 12.8 and 19.2 mmol NO_3_^−^ ingestion, while mean rate of torque development (RTD) at 0–50 ms and 0–100 ms was enhanced following 6.4 mmol NO_3_^−^.
30	Williams T.D. et al. [49]	11 men	Beetroot juice -70 mL containing ~400 mg NO_3_^−^; 2 h before exercise, within 5 min or PLA (blackcurrat juice).	Juice	Acute	Acute BRJ supplementation increased mean velocity and mean power during free-weight bench press compared to placebo. Total repetitions across 3 sets to failure at 70% 1 RM (repetition maximum) increased. Set-to-set declines in repetitions were observed for both conditions (BRJ and PLA), but total repetitions were higher with BRJ. Overall, BRJ enhanced upper-body muscular strength, endurance and power output.
31	Wong T.H. et al. [74]	17 men	Two trials—2 × 285 mL of either ISO-BR (isotonic beetroot juice) or BR drink 3 h before testing Both contained 6.45 mmol, 400 mg, per 285 mL serving; 9 mg/100 mL of ascorbic acid was added into the ISO-BR drink.	Juice	Acute	ISO-BR increased salivary NOx more than BR and improved peak power output. Rate of fatigue was higher (+7.9%) with ISO-BR. Plasma NOx increased similarly for both drinks (~4-fold), and salivary NOx strongly correlated with plasma NOx.
32	Yuschen X. et al. [75]	12 men	2.5 h before exercise- Nitrate-rich beetroot juice (NRBRJ): 70 mL shot 400 containing 400 mg NO_3_^−^ or PLA (prune juice).	Juice	Acute	Mean exercise load increased with NRBRJ. Diastolic blood pressure (DBP) decreased at rest and at 5, 20, and 30 min during exercise. Mean arterial pressure (MAP) decreased at rest and at 20 and 30 min during exercise. Brachial–ankle pulse wave velocity (baPWV) decreased after exercise. Flow-mediated dilation (FMD) increased before and after exercise compared to PLA.

BRJ/BJ/BR—beetroot juice; PLA/PL—placebo; CON—control condition; NRBRJ—nitrate-rich beetroot juice; ISO-BR—isotonic beetroot juice; NO_3_^−^—nitrate ion; NO_2_^−^—nitrite ion; NO—nitric oxide; NOx—combined nitrate + nitrite concentration; KNO_3_—potassium nitrate; VO_2_—oxygen consumption; VO_2_max—maximal oxygen uptake; HR—heart rate; BP—blood pressure; DBP—diastolic blood pressure; SBP—systolic blood pressure; MAP—mean arterial pressure; baPWV—brachial–ankle pulse wave velocity; FMD—flow-mediated dilation; PetCO_2_—end-tidal carbon dioxide pressure; VCO_2_—carbon dioxide production; VE—minute ventilation; VO_2_—ventilatory equivalent for oxygen; VCO_2_—ventilatory equivalent for carbon dioxide; RER—respiratory exchange ratio; ΔHHb—change in deoxyhemoglobin + deoxymyoglobin; RMSSD—root mean square of successive differences; HRV—heart rate variability; RTD—rate of torque development; CMJ—countermovement jump; RTF—repetitions to failure; MP—mean power; PP—peak power; CON/ECC—concentric/eccentric muscle contractions; FS—full squat; WOD—workout of the day; WAnT—Wingate anaerobic test; IR1/Yo-Yo IR1—Yo-Yo Intermittent Recovery Test; 1RM—one-repetition maximum; TTE—time to exhaustion; RPE—rating of perceived exertion; TQR—total quality recovery; MLSI—medial–lateral stability index; OSI—overall stability index; APSI—anterior–posterior stability index; HC—hormonal contraceptives; GPS—global positioning system; CAF—caffeine; CIT—citrulline; POM—pomegranate powder; NAC—N-acetylcysteine; MAL—maltodextrin.

**Table 3:** Summary of the original studies examining the impact of dietary nitrates from beetroot on exercise performance—chronic dosing.

	Positive Effects of Supplementation (Chronic)					
	Study	Sample size	Dose	Form	Duration	Results
1	Burgos J. et al. [76]	32 men	I-placebo group (PLG); II-CIT (citrulline) group (CITG): 3 g/day (3 × 1 g) gelatin capsules III-nitrate-rich beetroot extract group (BRG); 3 gelatin capsules of 700 mg a day (5:1 beetroot extract equivalent to 3500 mg of whole dried root, standardized to contain 0.3% betanin providing 100 mg of NO_3_^−^) IV-CIT-BR group (CIT-BRG).	Capsules	Chronic (9 weeks)	CIT-BRG prevented increases in cortisol and decline in testosterone/cortisol ratio (T/C) compared to PLG and showed significantly higher % change in T/C vs. PLG and CITG (*p* < 0.05). CITG and BRG alone showed decreases in testosterone levels;
2	Burgos J. et al. [77]	32 men	6 capsules/day: (I) placebo group (PLG); (II) CIT (citrulline) group (3 × 1 g CIT; CITG); (III): nitrate-rich beetroot extract group (3 × 700 mg; 100 mg of NO_3_^−^; BRG) and (IV) CIT-BR group (CIT- BRG)	Capsules	Chronic (9 weeks)	Nine weeks of CIT + BR supplementation (CIT-BRG): significant improvements in aerobic power compared to PL and CIT alone. CIT-BRG also showed significant increases in maximal strength (HJUMP) and endurance-strength (1-MAT) after 9 weeks, whereas the other groups showed smaller or no improvements. Handgrip dynamometer (DYN) performance increased in all groups, but CIT-BRG displayed higher percentage improvements.
3	Daab W. et al. [36]	13 men	Beetroot juice (BET; 150 mL per serving, 250 mg NO_3_^−^/serving), consumed twice daily (08:00 and 18:00) for 7 consecutive days, including 3 days pre-exercise, on the trial day, and 3 days post-exercise or PLA (nonspecified)	Juice	Chronic (7-days)	7 days of high-nitrate BET improved recovery of muscle function and reduced perceived muscle soreness after simulated soccer match, without affecting blood markers of muscle damage.
4	de Oliveira G.V. et al. [78]	14 men	100 g of beetroot-based nutritional gel (BG; 12.2 ± 0.2 mmol of nitrate); On the second and third visit: a single dose of BG after measuring maximal forearm muscle isometric strength; Then 120 min before exercise, ingestion of the supplement; or PLA (nitrate-depleted BG gel).	Gel	Chronic, 8-day supplementation	High-nitrate beetroot gel (BG) supplementation significantly attenuated the decline in handgrip strength after exercise. A single dose of BG improved maximal forearm isometric strength recovery 20 min post-handgrip exercise.
5	Esen O. et al. [79]	14 men	2 × 70 mL/day (~12.8 mmol/day NO_3_^−^) for 5 days; on the experimental trial day, both shots were taken together 2.5 h before testing; or PLA (nitrate-depleted beetroot juice).	Juice	Chronic (5-day supplementation) with acute dosing on the test day	Short-term NO_3_^−^ supplementation reduced motor unit potential (MUP) duration during brief isometric contractions and recovery stages with and without blood flow restriction.
6	Esen O. et al. [80]	14 men	NO_3_^−^-rich beetroot juice (BRJ; NIT: 2 × 70 mL/day, ~12.8 mmol/day NO_3_^−^) or NO_3_^−^-depleted BRJ as placebo (PLA; 2 × 70 mL/day, ~0.08 mmol/day NO_3_^−^). For days 1–4, doses were taken morning (~9 a.m.) and evening (~9 p.m.); on day 5, both doses were taken together 2.5 h before exercise testing.		Chronic (2 × 5-days)	Chronic NO_3_^−^ supplementation (~12.8 mmol/day) elevated plasma NO_2_^−^, reduced BP at rest, and attenuated BP increases during short and sustained isometric muscle contractions.
7	Esen O. et al. [81]	10 men and 6 women	Nitrate-rich (NIT) beetroot juice 2 × 70 mL/day (~12.8 mmol/day NO_3_^−^) for 4 days (morning & evening), plus 2 × 70 mL 2.5 h before trial; or PLA (nitrate-depleted beetroot juice).	Juice	Chronic (short term; 2 × 5-days separated by a washout period)	Plasma [NO_2_^−^] increased by ~140% in NIT vs. PLA (*p* < 0.001). Motor unit potential (MUP) duration was significantly shorter in NIT vs. PLA during brief and sustained contractions with BFR.
8	Huang X. et al. [32]	44 men and 36 women	Concentrated beetroot juice (BRJ; 6.5 mmol NO_3_^−^/70 mL) or nitrate-free placebo (PL; 0.065 mmol NO_3_^−^/70 mL), 3 × 70 mL/day for 7 days.	Juice	Chronic (7-days)	Running economy: Lower mean VO_2_, RER, and blood lactate (BLA) during submaximal treadmill running at high speed (V3: males 13.3 km/h, females 11.6 km/h) in BRJ vs. PL. Time-to-exhaustion (TTE) during cycling at 85% peak power output (PPO): Significantly increased in BRJ vs. PL (male: 16.50 ± 3.09 min vs. 14.42 ± 2.62 min, *p* = 0.02; female: 12.38 ± 2.23 min vs. 10.86 ± 2.21 min, *p* = 0.04). Rating of perceived exertion (RPE): Lower with BRJ, though differences were not statistically significant. One week of BRJ supplementation improved submaximal running economy and cycling endurance (TTE) but did not enhance cross-country skiing performance; RPE tended to decrease but not significantly.
9	Khosravi S. et al. [51]	12 men	Beetroot juice (BRJ), 2 × 70 mL/day (~12.8 mmol NO_3_^−^ per day) for 6 days; exercise testing on day 6, 2–2.5 h after the final dose; or PLA (blackcurrant juice).	Juice	Chronic; (6 days)	Six days of BRJ supplementation increased peak torque during bilateral isokinetic knee extensions. Improvements were observed at high angular velocities: 180 and 360°/s for the dominant leg and 360°/s for the non-dominant leg. BRJ also enhanced muscle fatigue resistance during 50 maximal knee extensions at 180°/s, with an increase in peak torque.
10	Kozłowska L. et al. [82]	10 men and 10 women	Freeze-dried beetroot juice (BRJ), 26 g/day (~200 mL juice equivalent, ~2.1 mmol NO_3_^−^), taken once daily with a meal 2 h before VO_2_max testing; or PLA (ID- dietary recommendations without additional BRJ).	Freeze-dried juice	Chronic (4-weeks)	VO_2_max significantly increased after BRJ supplementation. Muscle damage marker LDH stabilized after BRJ supplementation. Serum creatine kinase (CK) activity was consistently higher in men than women across all stages. Oxidative stress marker malondialdehyde concentration (MDA) increased after BRJ, while antioxidant defense markers (selenium, glutathione peroxidase activity- GPx1, GPx3) increased.
11	Liubertas T. et al. [83]	13 men	Oat bar (60 g; 4 g standardized *Amaranthus hypochondriacus* concentrate; ≈400 mg NO_3_^−^), consumed 1 h before exercise during single-dose testing, and daily for 6 days before long-term testing; or PLA (60 g-oat bar with excluded *Amaranthus hypochondriacus*).	Oat bar	Chronic (6 days) and single-dose test performed 1 h after first ingestion	Increase of peak power of increasing cycling exercise (ICE); Long-term use of dietary amaranth: VO_2_max demonstrated a significant increase.
12	Nicholas C. et al. [84]	10 men	140 mL/day of NO_3_^−^−-rich (12.8 mmol·d^−1^; BRJ + lemon juice); 2.5 h before the trial; or PLA (nitrate-depleted BRJ + lemon juice for taste).	Juice	Chronic (6 days)	Improved heavy load carriage; Increased heart rate, mean tidal volume, and performance during time trial with BRJ.
13	Rowland S.N. et al. [85]	9 men	Beetroot powder—NO_3_^−^-rich (BR, 6% NO_3_^−^, 8 mmol NO_3_^−^). Participants consumed 8.4 g/day in ≥250 mL water for 6 days. On day 7, a pre-exercise dose 2 h before cycling and a top-up 8.4 g dose 1 h into the 2-h exercise.	Powder dissolved in water	Chronic (Two 7-day supplementation periods (BR or PL), cross-over, with experimental testing on day 7 including pre- and mid-exercise top-up doses)	Short-term nitrate-rich beetroot powder supplementation increased plasma [NO_3_^−^] and [NO_2_^−^], improved end-sprint mean power output, and enhanced muscle oxygenation (deoxyhaemoglobin + deoxymyooglobin concentration [HHb] (kinetics)).
14	Tan R. et al. [86]	8 men and 4 women	Nitrate-rich beetroot juice (BR, ~6.2 mmol NO_3_^−^− per 70 mL, 2 × 70 mL/day) compared to NO_3_^−^-depleted beetroot juice placebo (PL, ~0.04 mmol NO_3_^−^− per 70 mL) and control water (CON); (Three separate 4-day supplementation periods (2 × 70 mL/day; days 1–2 one morning + one evening, days 3–4 both in morning ~2.5 h before exercise).	Juice	Chronic	BR supplementation significantly increased plasma [NO_3_^−^] and [NO_2_^−^] compared to PL and CON. During moderate-intensity cycling, BR reduced the O_2_ cost of exercise by ~2% when ensemble-averaged over four-step exercise transitions
15	Tirkey D. et al. [33]	15 men and 15 women	Beetroot juice (BRJ) 250 mL/day in natura, providing ~5.00 mmol NO_3_^−^ per day; or PLA (nitrate-depleted beverage).	Juice	Chronic (15 days)	Fifteen days of daily BRJ supplementation improved 10-km running time trial performance in both male and female trained athletes. Male experimental group showed a 4.2% improvement. Female experimental group showed a 7.5% improvement.
16	Viribay A. et al. [87]	20 men	Per day: (I) 5 capsules of placebo and 6 g of maltodextrin in powder; (II) 5 capsules (500 mg) of BR and 6 g of maltodextrin in powder; (III) 5 capsules of BR (500 mg) and 6 g of CIT in powder.	Capsules	Chronic (7-days)	7 days of combined BR extract and CIT supplementation enhances lactate clearance after high-intensity exercise and improves peak power during aerobic testing in elite rowers, providing modest ergogenic benefits.

BRJ/BJ/BR—beetroot juice; PLA/PL—placebo; CON—control condition; NRBRJ—nitrate-rich beetroot juice; ISO-BR—isotonic beetroot juice; NO_3_^−^—nitrate ion; NO_2_^−^—nitrite ion; NO—nitric oxide; NOx—combined nitrate + nitrite concentration; KNO_3_—potassium nitrate; VO_2_—oxygen consumption; VO_2_max—maximal oxygen uptake; HR—heart rate; BP—blood pressure; DBP—diastolic blood pressure; SBP—systolic blood pressure; MAP—mean arterial pressure; baPWV—brachial–ankle pulse wave velocity; FMD—flow-mediated dilation; PetCO_2_—end-tidal carbon dioxide pressure; VCO_2_—carbon dioxide production; VE—minute ventilation; VO_2_—ventilatory equivalent for oxygen; VCO_2_—ventilatory equivalent for carbon dioxide; RER—respiratory exchange ratio; ΔHHb—change in deoxyhemoglobin + deoxymyoglobin; RMSSD—root mean square of successive differences; HRV—heart rate variability; RTD—rate of torque development; CMJ—countermovement jump; RTF—repetitions to failure; MP—mean power; PP—peak power; CON/ECC—concentric/eccentric muscle contractions; FS—full squat; WOD—workout of the day; WAnT—Wingate anaerobic test; IR1/Yo-Yo IR1—Yo-Yo Intermittent Recovery Test; 1RM—one-repetition maximum; TTE—time to exhaustion; RPE—rating of perceived exertion; TQR—total quality recovery; MLSI—medial–lateral stability index; OSI—overall stability index; APSI—anterior–posterior stability index; HC—hormonal contraceptives; GPS—global positioning system; CAF—caffeine; CIT—citrulline; POM—pomegranate powder; NAC—N-acetylcysteine; MAL—maltodextrin.

**Table 4:** Summary of the original studies examining the impact of dietary nitrates from beetroot on exercise performance.

	Non-Significant/No Effects of Supplementation					
	Study	Sample Size	Dose	Form	Duration	Results
1	Berjisian, E. et al. [88]	16 men	One 60-mL bottle of fluid containing either 6.4 mmol (NO_3_^−^), 500 mg L-Arginine, and L-Ornithine or NO_3_^−^ depleted dried powder as placebo and ingested a capsule containing 5 mg/kg body mass of caffeine (CAF) or cellulose as PL 60 min before the start of the Stroop test. Four experimental trials: BJ + CAF, CAF + PL, BJ + PL, and PL + PL.	Juice	Acute	No significant effect of BJ, CAF, or BJ + CAF on total distance covered (YYIR1 test (intermittent running) No significant effect of supplementation on CMJ height and power. RPE increased over time; no supplementation effect. No supplementation effect on cognitive performance.
2	Berlanga L.A. et al. [89]	10 men	150 min before testing: 70-mL dose of BJ (6.4 mmol of NO_3_^−^); or PLA (nitrate-depleted BJ).	Juice	Acute	Acute ingestion of 70 mL beetroot juice (6.4 mmol NO_3_^−^) did not improve neuromuscular performance.
3	Burke L.M. et al. [38]	21 men	Study 1: two evenings before the experimental trial (−36, and −12 h): 70 mL shot of NO_3_^−^-rich beetroot juice (BRJ; 6.45 mmol NO_3_^−^); Morning of the experimental trial: 140 mL (~12.9 mmol NO_3_^−^) of BRJ supplement with breakfast+ second treatment after 7 km exercise: 70 mL BRJ (6.45 mmol NO_3_^−^); after each treadmill 26-km protocol: 190 mL of allocated test drink Study 2: Carb Max	Juice	Acute	Two-day preload plus pre- and mid-exercise ingestion of beetroot juice increased plasma nitrate concentrations throughout the ~2 h exercise session. Plasma nitrite concentrations were elevated only during the first half of exercise; levels declined in the second half. Beetroot juice did not alter oxygen uptake or the oxygen cost of exercise at race-relevant speeds. Beetroot juice supplementation did not improve endurance performance, exercise efficiency, or oxygen utilization in elite race walkers
4	Collins S.M. et al. [90]	15 men and 9 women	Subjects performed two counterbalanced trials, once with a control and another after consuming 70 mL (~4.2 mmol NO_3_^−^) of beetroot concentrate nitrate supplement 2 h prior to physical activity; or PLA (strongly flavored water).	Beetroot concentrate	Acute	Acute ingestion of 70 mL beetroot nitrate did not improve high-intensity functional training (HIFT) performance, lactate or perceived exertion.
5	Conger S.A. et al. [91]	14 men	The supplement was provided to the participant 24 to 72 h preceding the trial. One dose of red beet juice powder containing ~8 mmol (496 mg) NO_3_^−^ mixed with 237 mL of water (this dose is considered “high” (high > 7.5 mmol); or PLA (cherry-apple-cranberry juice blend).	Juice	Acute	Beet juice did not enhance overall power output, endurance, or performance in short-term maximal anaerobic cycling; minor reduction in fatigue was only observed in the 30-s test.
6	Esen O. et al. [92]	12 men	140 mL NO_3_^−^-rich (BRJ; 2 × 70 mL; ~12.8 mmol NO_3_^−^) or NO_3_^−^-depleted (PLA) BRJ, 3 h before two experimental trials three.	Juice	Acute	Acute high-dose BRJ (12.8 mmol NO_3_^−^) increases plasma NO_3_^−^/NO_2_^−^ but does not improve intermittent running performance, CMJ (counter-movement jump), or blood lactate responses in trained male rugby players.
7	Fernández-Elías V. et al. [45]	9 men	3 h prior to exercise: 70 mL of concentrated beetroot juice (6.4 mmol NO_3_^−^); or PLA (0.005 mmol of NO_3_^−^) prepared by dissolving 1 g of powdered beetroot and lemon juice in water.	Juice	Acute	Acute ingestion of nitrate-rich beetroot juice did not increase total distance covered, running speed, or high-intensity running during a 3-set tennis match. Serve speed before and after the match was unchanged between beetroot juice and placebo conditions. Isometric handgrip strength before and after the match showed no differences between treatments. RPE post-match was not affected by beetroot juice ingestion. Beetroot juice did not improve pre-to-post match changes in tennis-specific performance measures.
8	Hennis P.J. et al. [93]	21 men and 6 women	3 days prior to exercise trials and continued throughout the exercise trials: 3 × 200 mL, daily nitrate consumption of ~0.18 [18.5 (±2.0) mmol]; or PLA (nitrate-depleted beetroot/fruit juice [1.4 (0.1) mmol]).	Juice	Chronic	Dietary nitrate did not alter exercise efficiency, anaerobic threshold, peak work rate, heart rate, or ventilation. Plasma nitrate, nitrite, and nitroso product concentrations were largely unchanged during exercise tests, with some post-exercise spikes in nitrite and S-nitrosothiol in a few individuals.
9	López-Samanes Á. et al. [39]	11 women	3 h before each testing session: 70 mL dose of beetroot juice (6.4 mmol of NO_3_^−^); or PLA (nitrate-depleted beetroot juice).	Juice	Acute	No effects of acute beetroot juice on countermovement jump height, isometric handgrip strength, 20 m sprint, repeated sprint ability, GPS-measured match-play activity, or perceived exertion in elite female field hockey players.
10	López-Samanes Á. [94]	13 men	Two separate occasions: 3 h before testing; 70 mL of either BJ (containing 6.4 mmol of NO_3_^−^) or PLA; (in each trial, 50% of participants ingested PLA and 50% ingested BJ beverages) with random assignment to each supplement.	Juice	Acute	Acute ingestion of 70 mL beetroot juice (BJ, 4.2–6.4 mmol NO_3_^−^) did not improve tennis-specific neuromuscular performance. No effects were observed on serve velocity, countermovement jump height, isometric handgrip strength, 5-0-5 agility, or 10 m sprint speed. RPE was unaffected. Low doses of nitrate precursors do not provide ergogenic benefits for highly trained tennis players.
11	Moreno B. et al. [95]	6 women and 7 men	Beetroot juice (BJ, 70 mL, 6.4 mmol NO_3_^−^) or nitrate-depleted placebo (PLA, 70 mL), ingested 3 h before swimming test. Two sessions separated by 18-day washout.	Juice	Acute	100-m times showed no difference between the BJ and (possible shorter time for BJ in the last repetition); BJ—possibly lower RPE in the first and second repetition; Total Quality Recovery scale scores likely higher in the first and third repetition (than in PLA). No difference in blood lactate concentration between; an increase in 100-m times for both BJ and PLA after the fifth repetition. No significant differences in performance in a 6 × 100-m repeated sprint test. Possible trend toward a better recovery and a better tolerance of fatigue after supplementing BJ.
12	Ortiz de Zevallos J. et al. [96]	12 women and 14 men	70 mL of beetroot juice (BRJ ~6.5 mmol NO_3_^−^) twice/day (~13 mmol total NO_3_^−^) for ~3 days or an identical NO_3_^−^-depleted placebo (PL). On testing days—the last two 70 mL shots 2 h before their laboratory arrival time. Female subjects were given additional bottles and were instructed to start consuming the juice the day before the estimated day of menses to consider any changes in the start of the menstrual cycle and guarantee consumption of at least 3 days of supplementation before experimental visits.	Juice	Acute (3-days)	Plasma NO_3_^−^ and NO_2_^−^ increased after supplementation. Cognitive tests showed no significant changes. Submaximal exercise economy and time-to-exhaustion improved only in males, not females. Muscular endurance, jump height and low power improved/increased only in some cases. No improvement in sprinting, high-load strength, and intermittent running. Post-exercise lactate clearance improved with some BR + CIT supplementation.
13	Robinson G.P. et al. [47]	8 men	3 h prior to testing: 140 mL of beetroot juice (providing ~12.4 mmol NO_3_^−^) daily for 7 days. On nonexperimental days (days 1–2, 4, and 6)—1 × 70 mL in the morning (~09:00) and 1 × 70 mL in the evening (~19:00); or PLA (~0.08 mmol NO_3_^−^).	Juice	Chronic	Beetroot juice (BR) supplementation increased plasma NO_2_^−^, providing more substrate for O_2_-independent nitric oxide synthesis during exercise but BR did not improve high-intensity intermittent running performance in endurance-trained males. Effects were consistent in normoxia and low-to-moderate hypoxia (~1200–2400 m). Overall, BR does not enhance high-intensity intermittent running performance in trained endurance athletes under these conditions.
14	Rokkedal-Lausch T. et al. [46]	12 well-trained cyclists (gender not specified)	140 mL of concentrated beetroot juice (~12.4 mmol nitrate) per day; one dose (70 mL) in the morning and one dose (70 mL) in the evening. On the days of the experimental trials: total dose 2-h before arriving at the laboratory; or PLA (nitrate-depleted BR).	Juice	Chronic (7 days)	BR supplementation did not alter oxygen uptake (VO_2_) or muscle deoxygenation (ΔHHb) kinetics during moderate-intensity cycling (~60–62% VO_2_max). BR reduced the amplitude of the VO_2_ response (~2.1%), but steady-state VO_2_, exercise efficiency, and steady-state ΔHHb remained unchanged. Effects of BR were similar in normoxia and hypoxia; no supplementation-by-condition interactions were observed. Hypoxia increased heart rate, carbon dioxide production (VCO_2_), minute ventilation (VE), and respiratory exchange ratio (RER), and reduced VO_2_ time delay, independently of BR supplementation.
15	Sousa A. et al. [97]	30 men	Three experimental groups: (I) HNO: high-intensity exercise training sessions in normobaric hypoxia with NO_3_^−^ supplement; (II) HPL: high-intensity exercise training sessions in normobaric hypoxia with placebo and (III) CON: high-intensity exercise training sessions in normoxia with placebo. Supplements given 2.5–3 h prior to each session. ((NO_3_^−^ beetroot juice; 400 mg of a powdered standardized beetroot extract (2% of NO_3_^−^, ~8.4 mmol))	Juice	Chronic	NO_3_^−^ supplementation did not enhance exercise performance at simulated altitude compared to placebo.
16	Tan R. et al. [98]	16 men	Four supplementation conditions: (1) PL with MAL (PL + MAL), (2) PL with NAC (PL + NAC), (3) BR with MAL (BR + MAL) (4) BR with NAC (BR + NAC); 2 × 70 mL doses per day of either BR (~6.2 mmol of NO_3_^−^per 70 mL) or PL. On day 1–5: one 70 mL beverage in the morning and one in the evening. On the experimental day: 2 × 70 mL of allocated beverage 2.5 h prior to exercise and 70 mg/kg of NAC (N-acetylcysteine; 600 mg NAC per capsule)) or maltodextrin (MAL; 600 mg per capsule) 1 h prior to exercise.	Juice	Chronic (6 days)	Muscle excitability decline during 1 h cycling was attenuated by NAC (PL + NAC) but not by BR or BR + NAC. Time to exhaustion (TTE) during severe-intensity cycling was not affected by BR, NAC, or their combination. Voluntary muscle activation and neural drive were unchanged across conditions. Co-ingestion of BR and NAC maintained plasma NO_3_^−^ but did not enhance endurance or performance.
17	Tan R. et al. [99]	15 men	(1) PL; (2) NO_3_^−^-rich beetroot juice (BR: ~12 mmol of NO_3_^−^) with 2 empty gelatin capsules; (3) BR with 2 capsules with pomegranate powder (POM: 1000 mg; BR + POM; On experimental: 2 × 70 mL of allocated beverage and capsules 2.5 h before exercise + on a separate visit: two capsules containing 1000 mg of POM 2.5 h prior to a blood draw	Juice + Capsules	Acute	POM did not increase plasma [NO_3_^−^] and [NO_2_^−^] compared to PL. BR + POM did not alter plasma [NO_3_^−^] and [NO_2_^−^] compared to BR. BR did not affect performance in vertical CMJ, explosive push-ups, or back squats.
18	Tan R. et al. [44]	15 women	2 × 70 mL of concentrated NO_3_^−^-depleted placebo (PL; 0.10 mmol NO_3_^−^ total) or NO_3_^−^-rich beetroot juice (BR; ~ 12.0 mmol NO_3_^−^ total) with a washout-out period of at least 5 days separating the two supplementation periods.	Juice	Acute	Acute nitrate-rich beetroot juice did not improve sprinting, strength, or aerobic performance in female team-sport players.
19	Trexler E.T. et al. [40]	27 men	2 h before exercise: (1) 70-mL beetroot juice beverage (400 mg dietary nitrate); (2) placebo (PLA); (3) 8 g of unflavored citrulline malate (CitMal)	Juice	Acute	A single dose of beetroot juice or CitMal does not improve physical performance, endurance, or exercise efficiency in resistance performance.
20	Viribay A. [100]	20 men	(I) Placebo group (PLAG); (II) Beetroot extract group (BRG); and (III) BR supplemented with L-citrulline group (BR-CITG). The intervention spanned 3 consecutive weeks, with each week corresponding to a distinct supplement-intake condition. Daily dosages for 7 days: (I) five placebo capsules per, alongside 6 g of maltodextrin powder; (II) five capsules (each containing 500 mg) per day of BR accompanied by 6 g of maltodextrin powder; or (III) 5 capsules per day (each containing 500 mg) of BR alongside 6 g of L-citrulline powder.	Capsules	Chronic (7-days)	7 days of co-ingestion of BR extract and CIT (citrulline) did not enhance physiological or metabolic outcomes during submaximal or maximal rowing exercise.

BRJ/BJ/BR—beetroot juice; PLA/PL—placebo; CON—control condition; NRBRJ—nitrate-rich beetroot juice; ISO-BR—isotonic beetroot juice; NO_3_^−^—nitrate ion; NO_2_^−^—nitrite ion; NO—nitric oxide; NOx—combined nitrate + nitrite concentration; KNO_3_—potassium nitrate; VO_2_—oxygen consumption; VO_2_max—maximal oxygen uptake; HR—heart rate; BP—blood pressure; DBP—diastolic blood pressure; SBP—systolic blood pressure; MAP—mean arterial pressure; baPWV—brachial–ankle pulse wave velocity; FMD—flow-mediated dilation; PetCO_2_—end-tidal carbon dioxide pressure; VCO_2_—carbon dioxide production; VE—minute ventilation; VO_2_—ventilatory equivalent for oxygen; VCO_2_—ventilatory equivalent for carbon dioxide; RER—respiratory exchange ratio; ΔHHb—change in deoxyhemoglobin + deoxymyoglobin; RMSSD—root mean square of successive differences; HRV—heart rate variability; RTD—rate of torque development; CMJ—countermovement jump; RTF—repetitions to failure; MP—mean power; PP—peak power; CON/ECC—concentric/eccentric muscle contractions; FS—full squat; WOD—workout of the day; WAnT—Wingate anaerobic test; IR1/Yo-Yo IR1—Yo-Yo Intermittent Recovery Test; 1RM—one-repetition maximum; TTE—time to exhaustion; RPE—rating of perceived exertion; TQR—total quality recovery; MLSI—medial–lateral stability index; OSI—overall stability index; APSI—anterior–posterior stability index; HC—hormonal contraceptives; GPS—global positioning system; CAF—caffeine; CIT—citrulline; POM—pomegranate powder; NAC—N-acetylcysteine; MAL—maltodextrin.

**Table 5:** Summary of the review articles and meta-analyses on the impact of dietary nitrates from beetroot on exercise performance.

	Review Articles		
	Study	Included Articles	Conclusions
1	Abreu R. et al. [101]	1 out of 18 included studies was about nitrate supplementation from beets; semi-professional soccer players were given nitrate-rich beetroot juice, simulated a soccer match, and their post-exercise performance was assessed;	Performance decreased after exercise in both groups, but the reduction was smaller with beetroot juice, suggesting possible benefits during long-term recovery.
2	Alsharif N. et al. [102]	27 studies—24 of them were describing supplementation of nitrogen coming from beetroot juice; Studies differed in the methods and duration of supplementation, age of participants, training levels, and sports disciplines.	It was noted that supplementation contributed to the improvement of: total distance covered, peak power, mean power output, total work done. The results from this review and meta-analysis confirm the ergogenic potential of dietary NO_3_ supplementation in some aspects of high-intensity exercise capacity
3	Antonio J. et al. [103]	5 studies about the supplementation of beetroot juice; the duration and method of administration (varied between the studies); examining parameters related to endurance and physical performance;	All five studies in the review reported benefits of beetroot supplementation, including increased time to exhaustion, reduced oxygen consumption, and improved training load. Beetroot juice supplementation by athletes may have a positive impact on their performance and physical endurance during training
4	Apte M. et al. [3]	10 studies assessing the effect of beetroot juice on physical performance; specifically time trial performance, 4 studies assessing the effect of beetroot juice on cognitive functions	Beetroot juice supplementation consistently improved time-trial performance across studies, with some evidence of cognitive benefits in young adults (18–30 years), though results were mixed. Natural dietary nitrates appear to be an accessible and low-cost ergogenic aid.
5	Calvo J. et al. [104]	27 studies—23 of them used beetroot as the supplemented nitrate source; studies varied in: duration and supplementation method, and the participants differed in their training levels and chosen sports.	Supplementation reduced VO_2_, improved pain threshold, and enhanced performance in sprint interval training, but heterogeneity requires more trials.
6	Chen L. et al. [105]	The review article discusses the health-promoting properties of beetroot, including its anti-cancer, antioxidant, kidney and liver protective properties, and its impact on physical performance.	Some studies showed gains in kayaking, resistance, and mountaineering performance, confirming the ergogenic potential of beetroot, though results remain varied.
7	Delleli S. et al. [106]	9 studies on athletes practicing various combat sports; (different age groups, different levels of training, supplementing beetroot in various forms, e.g., juice, capsules or gel)	In combat sports, six studies reported improved performance with beetroot supplementation, while three found no benefit or deterioration. Effectiveness may depend on training level, and further confirmation is required.
8	Domínguez R. et al. [107]	The aim of the review was to discuss the state of knowledge about nutrition and dietary practices in tennis players in order to maximize sports performance	Beetroot juice supplementation improved agility and handgrip strength in tennis players and improved performance in endurance, high-intensity sports and resistance training.
9	d’Unienville N.M.A. et al. [48]	meta-analysis included a total of 118 studies; 56 studies were related to nitrates from beetroot, red spinach, Swiss chard and rhubarb.	Nitrate supplementation showed benefits only when derived from beetroot, especially in less trained individuals, while no clear effects were observed in women. Sex differences and limited data in female athletes highlight the need for more targeted research.
10	Esen O. et al. [108]	19 studies on beetroot juice (the chosen placebo varied across studies); Age, athletic ability, and disciplines varied significantly among the included studies. The duration of administration and supplementation doses were inconsistent;	Beetroot juice slightly enhanced peak and mean power and time to peak power but showed no effect on isometric strength. Wide variability among studies limits practical interpretation.
11	Gamonales J.M. et al. [109]	15 studies; 14 of them administered nitrates in the form of beetroot juice; these studies were characterized by significant heterogeneity in terms of dosing regimen, participant characteristics, and the parameters studied;	Some studies reported benefits in jump performance, pain threshold, and VO_2_ recovery, while five found no differences. Effects appear small, inconsistent, and require further exploration. Over 60% indicated positive effects on regeneration, but evidence remains heterogeneous.
12	Gilsanz L. et al. [110]	6 studies in which subjects of various stages of advancement and different ages were given beetroot juice, caffeine, their combination or placebo; sportsmen practiced various sport disciplines (e.g., cycling, triathlon, soccer)	No studies demonstrated improvements in physical performance parameters with beetroot juice compared to placebo.
13	Harlow J. et al. [111]	one study was included in the review; assessment of the effects of beetroot juice supplementation (by soldiers) on exercise capacity and high-altitude acclimatization;	Beetroot juice improved exercise performance compared with placebo and promoted faster post-exercise heart rate recovery. Beetroot juice may have a positive effect on performance during high-intensity, moderate-duration workouts.
14	Hogwood A. et al. [112]	11 studies, only 8 of them were conducted on healthy people	A small, non-significant trend toward improved VO_2_peak was observed, but overall effects were inconsistent. Adding beetroot juice to training did not enhance outcomes beyond exercise alone.
15	Jones L. et al. [113]	9 studies—8 of which administered nitrates in the form of beetroot juice; 6 of these studies were included in the meta-analysis; Included studies differed in protocols and supplementation regimens; Participants varied in age and athletic ability; no professional athletes were included;	Beetroot juice improved recovery of isometric strength and jumping ability but had no effect on oxidative stress markers. Benefits may depend on training modality.
16	Kiani A. et al. [114]	This review discusses various positive aspects associated with supplementation that increases blood nitrogen levels. Two studies were included that examined the effect of supplementation on improving physical performance in athletes.	Beetroot supplementation significantly improved completion time, average power output, and time to exhaustion in cycle ergometer time trials compared with placebo. These findings suggest benefits for high-intensity endurance exercise, though further studies are needed to define optimal supplementation strategies.
17	Kim J. et al. [115]	The review included studies that examined the effects of supplementation with caffeine, beta-alanine, sodium bicarbonate, β-hydroxy-β-methylbutyric acid, and beetroot juice. The latter included two studies in which well-trained rowers were given beetroot juice at varying doses and with different supplementation schedules.	Rowing studies demonstrated improved repetitions and 2000-m performance, particularly in moderately trained athletes, alongside rises in plasma nitrite levels.
18	Lago-Rodríguez Á. et al. [116]	5 studies—all involving 60 participants; 4 of these studies involved healthy individuals and both sexes from different age groups; The supplementation model differed in the administered dose of nitrates from beetroot juice.	Studies on healthy individuals found no effect on isokinetic torque but suggested potential benefits in less trained or short-term contexts. Evidence remains limited.
19	López-Laval I. et al. [117]	19 studies, two of which concerned beetroot supplementation in gel form, and the remaining studies concerned other nutrients. The volunteers studied varied in their discipline, age, supplementation regimen, and the parameters examined	Supplementation improved muscle oxygen saturation, recovery of strength, and reduced exercise-induced strength loss, though variability limits firm conclusions.
20	López-Torres O. et al. [118]	6 studies in which women were given beetroot juice and their athletic performance was assessed; In other 5 studies, women were given beetroot juice; various sports and different fitness levels;	Results were mixed in women and elite athletes, with no benefits reported in some groups, while kayakers and runners showed significant improvements. More research in women is particularly needed.
21	Mohd Daud S.M. et al. [119]	26 studies, 5 of which administered beetroot juice; different supplementation protocols and examined parameters between the studies	Supplementation improved muscle recovery in volunteers and also reduces post-exercise muscle pain. Fruit juices may be the best natural-based dietary supplements, replacing other supplement products in supporting muscle recovery and improving athletic performance in trained athletes. Future research, focusing on optimal dose, timing, and frequency of consumption, is needed.
22	O’Connor E. et al. [120]	5 studies that assessed the effect of this supplementation on post-exercise recovery. The athletes’ disciplines, their training levels, and the parameters studied and supplementation parameters varied between studies.	Supplementation reduced post-exercise muscle soreness in soccer players and sprinters, but not in endurance athletes. Blood markers of damage, oxidative stress, and inflammation were unaffected.
23	Poulios A. et al. [121]	one paper on long-term supplementation with beetroot juice in semi-professional football player; study examined vertical jump, speed and strength, as well as reduction of post-exercise muscle pain	Supplementation enhanced jump height, strength, speed, and reduced muscle soreness, but did not alter biochemical markers of muscle damage.
24	Rojano-Ortega D. et al. [122]	9 studies; Volunteers: were of different age groups, participated in a big of sports, and the supplementation regimen varied between the studies; The protocols for the parameters studied were also uniform	Four of these studies demonstrated improvement in these variables, four studies also demonstrated improvement in muscle soreness, and only one study demonstrated a significant difference in creatine kinase levels after beetroot supplementation versus placebo. However, no effect of supplementation on inflammatory markers was demonstrated.
25	San Juan A. et al. [123]	4 studies involving 49 men exercising at least twice a week; The studies differed in: duration, supplementation method, and the examined parameters. In 3 of the 4 included studies, volunteers were given nitrogen coming from beets.	All included studies showed gains in resistance training outcomes like repetitions, bench press power, and VO_2_ reduction. Beetroot may benefit both racquet sports and weightlifting, though mechanisms remain unclear.
26	Silva K. et al. [124]	Non-disclosed number of studies; only information about the number of participants; 168 participants, from included studies, received nitrates in the form of beetroot juice and 9 in the form of beetroot gel;	A meta-analysis concluded that beetroot juice is more effective than other nitrate sources, particularly for exercise lasting 2–10 min.
27	Tan R. et al. [125]	6 studies on cyclists performing sprints; Examining: average power, peak power, time to peak power and minimum power during 30-s sprints;	Supplementation positively influenced time to peak power during short sprints, but had no effect on average or peak power. Findings are promising but limited, requiring further research.
28	Tan R. et al. [126]	6 studies about nitrate-rich beetroot juice supplementation; the model and methods of supplementation in these studies were different, as were the methods of testing performance; studies were not standardized;	Supplementation improved repetitions to failure, average power, and velocity in resistance exercise. However, study heterogeneity limits the strength of conclusions.
29	Tan R. et al. [127]	18 studies—in 17 of them, the chosen form of supplement was beetroot juice; Training levels, age, methods of supplementation, tested parameters and sports disciplines, as well as the testing methods differed between the studies;	Positive effects were observed in squat, knee strength, and bench press velocity, but further standardized studies are needed to confirm findings.
30	Tanabe Y. et al. [128]	8 studies on beetroot juice supplementation; Different physical activities across the studies (including the disciplines and level of advancement) The study and supplementation protocols varied;	Some studies reported improvements in creatine kinase and faster recovery in strength and VO_2_, but no reductions in blood markers of muscle damage.
31	Vicente-Salar N. et al. [129]	6 studies on supplementing nitrates from beetroot in the form of juice, extract, or gel; The volunteers varied in terms of their sport, skill level, and age; The supplementation regimen and the parameters examined were also not identical in each study;	In combat sports, several studies found improved physical performance and reduced soreness, though no effects were seen on inflammation markers (only one study demonstrated a significant difference in creatine kinase levels). A supplement such as beetroot juice needs further research to strengthen the evidence of its positive effect in improving performance in combat sports and other disciplines.
32	Vicente-Salar N. et al. [130]	21 studies; only one study involved beetroot juice supplementation.	In elite tennis players, supplementation did not improve explosive movements or perceptual effort. Further research is needed to assess strength-related outcomes.
33	Wong T.H. et al. [131]	17 studies on supplementing nitrates in the form of beetroot; age, training level, method of supplementation and tested parameters differed between the studies;	Evidence was mixed: some studies showed improvements in power and performance whereas others showed no change or declines. Supplementation may help alleviate muscle soreness, but variability remains high.
34	Wong T.H. et al. [132]	24 studies—20 of them were about supplementing nitrogen coming from beets; studies differed in terms of protocol and method of supplementation, age and training levels of participants and practiced disciplines.	Time trial performance improved in cycling trials (4–5 km), with slightly faster completion after beetroot versus placebo.
35	Zamani H. et al. [133]	The impact of a single dose and the length of supplementation was assessed for men and women with different fitness levels.	A single dose improved blood flow, sprint and interval performance, time to exhaustion, and post-exercise recovery, particularly in less trained athletes.
36	Zoughaib W.S. et al. [134]	5 studies on nitrates derived from salt or beetroot juice; The study groups were of different ages and fitness level; 4 of the included studies involved healthy individuals;	Beetroot improved distance, power, and work done, and may be more effective than nitrate salts, though study numbers are small.

## 4. Discussion

Beetroot (*Beta vulgaris rubra*) has been a part of the human diet for centuries, originally valued for its pigments, fiber, vitamins, and minerals [135]. More recently, however, attention has shifted toward its high dietary nitrate (NO_3_^−^)content. Although the biological effects of nitrates have been recognized since antiquity—early records describe the use of potassium nitrate (KNO_3_) in ancient China to treat heart problems [136]—beetroot has only begun to be systematically examined as a natural source of nitrate in the past two decades, particularly after discoveries that dietary nitrate could lower blood pressure and improve vascular function in humans. Modern research began around 2007, when controlled trials showed that nitrate supplementation reduced the oxygen cost of submaximal exercise [137]. This has sparked widespread interest in beetroot as both a cardiovascular and performance-enhancing nutritional aid.

This systematic review examined studies published between 2020 and 2025 on beetroot supplementation and its effect on physical activity and cognitive functions.

In the late 1980s, researchers discovered that blood vessels release a substance called endothelium-derived relaxing factor (EDRF), which turned out to be nitric oxide (NO). They demonstrated that NO is made from an amino acid called L-arginine, and that drugs, such as nitroglycerin, exert their effects by releasing this “newly” identified molecule. This finding changed the view of nitrates and nitrites—from being seen only as chemicals or pollutants to being seen as important natural molecules that help regulate blood flow and vascular function [138].

For many years, dietary nitrate (NO_3_^−^) and nitrite (NO_2_^−^) were believed to play no significant physiological role in the body and were mainly associated with potential toxicity. It is now clear that both can act as alternative sources of nitric oxide (NO), especially when oxygen levels are low [139]. The physiological effects ofnitrates begin with the so called “enterosalivary mechanism”. Nitrates (NO_3_^−^) consumed with food are absorbed in the intestines and then secreted to saliva, where they are reduced to nitrite (NO_2_^−^). The final step is the conversion of nitrite to nitric oxide (NO), which occurs under conditions of hypoxia and low pH found in working muscles. This phenomenon is often observed during intense physical exercise [140,141].

The most important effect of nitric oxide, in terms of physical exercise, is the activation of guanylate cyclase in vascular muscles, which leads to an increase in cGMP concentration and, consequently, vasodilation. This increases the blood flow to the muscles, which allows a more efficient delivery of oxygen and energy substrates, and faster removal of exercise-related metabolites [142]. Another important mechanism involves the effects of NO and its derivatives on mitochondrial function. Research shows that nitrates increase the efficiency of oxidative phosphorylation and improve the ATP-to-oxygen ratio. This results in a lower oxygen cost, observed as a decrease in oxygen consumption under the same mechanical load [143].

Researchers have also shown that NO influences the muscle contractile apparatus. Its effect on calcium channels and proteins regulating Ca^2+^ release in the sarcoplasmic reticulum promotes more efficient energy utilization during contraction. Studies suggest that this effect is most pronounced in fast-twitch muscles, documenting improved performance during very high-intensity exercise with a predominance of anaerobic metabolism [144].

The effects of beetroot-derived nitrates on cognitive function remain unclear. Unlike in physical performance, where moderate doses show consistent benefits, there is no consensus regarding the nitrate dose or concentration most effective for improving executive function, attention, or memory. Further research using standardized cognitive tests is needed to determine whether moderate or prolonged supplementation can enhance brain function. Given the limited number of trials, systematic investigations are crucial to clarify how nitrates from beetroot juice affect cognition and to establish effective dosing strategies.

In recent years, inorganic (dietary) nitrates have been increasingly recognized as potential ergogenic agents due to their role in enhancing nitric oxide’s bioavailability. NO is a signaling molecule that relaxes blood vessels, allowing better blood flow and oxygen delivery to working muscles and reduces pulmonary vascular resistance, improving ventilation, perfusion and oxygen uptake. It also improves mitochondrial efficiency and reduces the oxygen cost of exercise, supporting endurance and recovery [18,143]. The multidirectional mechanisms of the physiological effects of nitrates on the human body discussed above suggest that they may result in an increased physical performance. Such effects have been observed in studies indicating a positive impact of beetroot supplementation on physical performance (Table 2 and Table 3).

Dietary nitrates in the most part (around 80% of dietary nitrate) come from vegetables. Vegetables with the greatest nitrate content (>1000 mg/kg) include are arugula, spinach, beetroot, lettuce, and celery. Nitrate levels are typically greater in leaves than in stems or roots. Vegetables with medium nitrate levels (100–1000 mg/kg) include cabbage, turnip, and green beans, while those with low levels (<100 mg/kg) include onions and tomatoes [137]. In the studies included in this paper, beetroot juice (BRJ) was the most commonly used form of nitrate supplementation. This vegetable has more practical advantages over other mentioned leafy greens, such as: it is easier to consume in effective amounts, is supported by a stronger research and provides other bioactive compounds (e.g., betalains, polyphenols). This supplement contains high concentrations of inorganic nitrates, and its liquid form facilitates precise dose determination and manipulation under controlled conditions [14,21]. An additional advantage of using juice supplementation is the possibility of concentrating it, which allows for the preparation of smaller, more convenient portions. The combination of elements such as rapid absorption, predictable pharmacokinetics, and extensive experimental control makes beetroot juice preferred in studies over powders, gels, or capsules.

Despite the large number of studies on this topic, findings on beetroot supplementation remain inconsistent. Several trials reported positive outcomes, such as improved muscular power and endurance time, reduced fatigue, better recovery, and in some cases enhanced VO_2_max or cardiovascular efficiency. The results of this re- view also show that there are studies on the basis of which it cannot be stated that supplementation with beetroot juice has a positive effect on the increasing body performance (Table 4). Ambiguous research outcomes may result, for instance, from different supplementation durations or varying experimental conditions. Mixed findings have also been reported for other nitrate-rich foods that may influence physical performance. Vegetables such as spinach, arugula, swiss chard and amaranth, have also been examined, though to a much smaller extent. Consistent with the findings from this review, one systematic review reported that, in randomized trials, red spinach extract improved several performance parameters, such as time, average power, relative power and average speed [145]. One study also tried to assess the effect of red spinach extract on cognitive functions, after a 7-day supplementation period. However, compared to placebo, the results were similar to studies included in this review, meaning red spinach extract supplementation had no significant effect on cognitive performance, nor subjective feelings of focus, energy, and fatigue [146].

Researchers have not only been investigating vegetables (also mentioned in this review), but also herbs have been given a closer look. One of the compounds with positive effects on exercise performance is caffeine. This plant-derived alkaloid has been proven to have positive ergogenic and cognitive effects. A study by Kovacs et al. showed that enhancement of endurance and anaerobic performance, similar to beetroot, can be achieved with doses between 2–9 mg/kg ~1 h pre-exercise [147]. Alongside positively affecting strength and endurance, based on reviewed articles, beetroot can lower blood pressure. Lowering blood pressure is also one of the effects that a plant called *Tribulus Terrestris* has on the human body [148]. However, this plant and beetroot share another similarity. Both have mixed results when it comes to such as: maximal strength or muscular endurance in resistance-trained men [149]. While nitrate appears to have ergogenic benefits, beetroot may show additional effects due to containing compounds such as betalains and polyphenols.

The present review identified only one study that examined the effects of nitrates on both physical performance and cognitive function [41]. As was previously revealed, these two elements are not only interconnected but also highly interdependent. Previous studies suggest that nitrates may affect both cognitive function and physical performance. Therefore, further studies should be conducted to examine the effects of nitrates on both parameters, so that clear conclusions can be drawn from a larger sample size and a variety of supplementation regimens, allowing creating optimal supplementation protocols.

Another limitation in drawing concrete conclusions and presenting the most favorable conditions for supplementation—its duration, administration schedule, and form—is the significant heterogeneity of the studies included in the review, with these parameters differing significantly. The studies often included individuals of varying ages, with significantly varying levels of training, and those practicing various sports. The studies lacked protocol consistency [106,108,110]. This problem, however, turns out to be quite common, as similar conclusions regarding the significant heterogeneity of studies documenting the effect of supplementation on physical performance were observed [150,151].

The above conclusions suggest that such research is necessary, as identifying the weaknesses of existing studies may support the development of more consistent future investigations which may lead to the discovery of new, interesting evidence on the benefits of supplementation to improve physical efficacy.

## 5. Summary and Conclusions

This literature review attempted to analyze existing evidence of the effects of nitrates, mostly derived from beetroot, on physical activity and cognitive functions and their influence on healthy aging. Evaluating physical activity and cognitive function in young to middle-aged adults is crucial, as this period represents the peak of physiological and cognitive performance as well as reproductive health and productivity. Sustaining regular physical activity during adulthood supports cardiovascular and metabolic health alongside musculoskeletal strength—key determinants of long-term health and disease prevention. What is more, preserving cognitive function enhances decision-making, and mental health, reducing the risk of neurodegenerative diseases later in life. Monitoring these factors promotes healthy aging and contributes to extending life expectancy. Research shows consistent improvements in cardiovascular health, endurance, and resistance exercise, especially in less trained individuals, usually supplementing beetroot juice in moderate doses of 6–12 mmol. However, findings in elite athletes remain inconsistent. Overall, beetroot supplementation shows strong potential as a natural ergogenic aid for physical performance. Evidence regarding cognitive improvement is still limited and inconclusive, proving the need for creating standardized protocols, larger trials, and detailed dose–response investigations. This gap in knowledge creates an opportunity for future researchers to examine the relationship between beetroot-derived nitrates and cognitive functions.

## Figures and Tables

**Table 1:** Search strategy in each database.

Database	Terms Combination
PubMed	((“Beetroot*”[Text Word] OR “beta vulgaris”[MeSH Terms] OR “beta vulgaris”[tw]) AND (“physical activit*”[Title/Abstract] OR physical[tw] OR “sport*”[Title/Abstract] OR “Athletic Performance”[MeSH Terms] OR “athletic performance*”[Title/Abstract] OR “exercise*”[Title/Abstract] OR “aerobic”[Text Word] OR “gymnastic*”[Text Word] OR “training”[Text Word]) AND “cognit*”[Text Word]) NOT (animals[mh] NOT humans[mh])
	((“Beetroot*”[Text Word] OR “beta vulgaris”[MeSH Terms] OR “beta vulgaris”[tw]) AND (“physical activit*”[Title/Abstract] OR “sport*”[Title/Abstract] OR “Athletic Performance”[MeSH Terms] OR “athletic performance*”[Title/Abstract] OR “exercise*”[Title/Abstract] OR “aerobic”[Text Word] OR “gymnastic*”[Text Word] OR “training”[Text Word])) NOT (animals[mh] NOT humans[mh])
Embase	(‘beetroot*’:ti,ab,kw,de,dn,df,mn,tn OR ‘beet’/exp OR ‘beet’ OR ‘beta vulgaris’:ti,ab,kw,de,dn,df,mn,tn) AND (‘physical activit*’:ti,ab,kw OR ‘physical’:ti,ab,kw,de,dn,df,mn,tn OR ‘sport*’:ti,ab,kw OR ‘athletic performance’/exp OR ‘athletic performance’ OR ‘athletic performance*’:ti,ab,kw OR ‘exercise*’:ti,ab,kw OR ‘aerobic’:ti,ab,kw,de,dn,df,mn,tn OR ‘gymnastic*’:ti,ab,kw,de,dn,df,mn,tn OR ‘training’:ti,ab,kw,de,dn,df,mn,tn) AND ‘cognit*’:ti,ab,kw,de,dn,df,mn,tn NOT ((‘animal’/exp OR ‘animal’) NOT (‘human’/exp OR ‘human’))
	‘beetroot*’:ti,ab,kw,de,dn,df,mn,tn OR ‘beet’/exp OR ‘beet’ OR ‘beta vulgaris’:ti,ab,kw,de,dn,df,mn,tn) AND (‘physical activit*’:ti,ab,kw OR ‘sport*’:ti,ab,kw OR ‘athletic performance’/exp OR ‘athletic performance’ OR ‘athletic performance*’:ti,ab,kw OR ‘exercise*’:ti,ab,kw OR ‘aerobic’:ti,ab,kw,de,dn,df,mn,tn OR ‘gymnastic*’:ti,ab,kw,de,dn,df,mn,tn OR ‘training’:ti,ab,kw,de,dn,df,mn,tn) NOT ((‘animal’/exp OR ‘animal’) NOT (‘human’/exp OR ‘human’))

* In database searches, the asterisk is used as a truncation symbol to substitute for zero or more characters, enabling retrieval of multiple word variants.

## Data Availability

Data sharing is not applicable (only appropriate if no new data is generated or the article describes entirely theoretical research).

## Supplementary Materials

The following supporting information can be downloaded at: https://www.mdpi.com/article/10.3390/nu17243954/s1, Table S1: PRISMA checklist.
